# Retrofitting ancient timber glulam mortise & tenon construction joints through computer-aided laser cutting

**DOI:** 10.1016/j.heliyon.2020.e03671

**Published:** 2020-04-11

**Authors:** Zhaoxi Zhan, Chen Wang, Jeffrey Boon Hui Yap, Ming Shyang Loi

**Affiliations:** a College of Civil Engineering, Huaqiao University, 361021, Xiamen, China; b Director of Intelligence and Automation in Construction Fujian Province Higher-educational Engineering Research Centre, College of Civil Engineering, Huaqiao University, 361021, Xiamen, China; c Department of Surveying, Lee Kong Chian Faculty of Engineering and Science, Universiti Tunku Abdul Rahman (UTAR), 43000, Kajang, Selangor, Malaysia; d Faculty of Built Environment, University of Malaya, 50603, Kuala Lumpur, Malaysia

**Keywords:** Architecture, Civil engineering, Materials science, Structural engineering, Computer-aided engineering, Tenon joints, Timber frame, Laser cutter, Mortise joints, Mortise & tenon joints

## Abstract

This study is aimed to rationalise and demonstrate the efficacy of utilising laser cutting technique in the fabrication of glulam mortise & tenon joints in timber frame. Trial-and-error experiments aided by laser cutter were conducted to produce 3D timber mortise & tenon joints models. The two main instruments used were 3D modelling software and the laser cutter TH 1390/6090. Plywood was chosen because it could produce smooth and accurate cut edges whereby the surface could remain crack-free, and it could increase stability due to its laminated nature. Google SketchUp was used for modelling and Laser CAD v7.52 was used to transfer the 3D models to the laser cutter because it is compatible with AI, BMP, PLT, DXF and DST templates. Four models were designed and fabricated in which the trial-and-error experiments proved laser cutting could speed up the manufacturing process with superb quality and high uniformity. Precision laser cutting supports easy automation, produces small heat-affected zone, minimises deformity, relatively quiet and produces low amount of waste. The LaserCAD could not process 3D images directly but needed 2D images to be transferred, so layering and unfolding works were therefore needed. This study revealed a significant potential of rapid manufacturability of mortise & tenon joints with high-quality and high-uniformity through computer-aided laser cutting technique for wide applications in the built environment.

## 1 Introduction

There were not many technological changes to the construction industry over the past 100 years even though there are many advancements to the construction technologies such as the emergence of 3D printing and laser cutting technology bringing imminent transformations to this industry (Kanyilmaz, 2019). For instance, users can now fabricate precise object swiftly employing a wide variety of digital fabrication tools such as 3D printers, laser cutters and milling machines (Mueller et al., 2012). Besides, automated digital manufacturing processes with advanced computer-aided design methods can be used to manufacture a large volume of products, prototypes and parts (Tobergte and Curtis, 2013). Laser cutting offers a high degree of precision, superior quality and speedy cutting of most materials using a non-contact, thermal-based fabrication process (Madic et al., 2012). Following the discovery of laser in the 1960s, the use of laser applications has been widespread, principally in material processing such as marking, cutting, engraving, drilling and welding of engineering structures to intensify accuracy and production quality (Riveiro et al., 2018). Laser beam cutting (LBC) is suitable for assorted types of materials, for example: wood, rubbers, plastics, ceramics and composites (Eltawahni et al., 2011). In the early 1970s, laser cutting of wood was introduced in the packing industry to cut plywood mouldings (Eltawahni et al., 2011). Thus, laser cutter allows intricate and precise patterns to be laser cut or engraved on various materials such as paper, textiles, card, glass and wood. In this regard, high-power engineering applications using a laser are seen to deliver improved cutting speeds, enhanced product quality and greater flexibility (Madic et al., 2012). Timber frame has a glorious history, whose origin could probably be traced back to the Roman Empire; used for centuries by woodworkers worldwide to bind pieces of woods in ancient timber houses (Mohamed, 2014). Indeed, the timber frame has existed for thousands of years and the mortise & tenon joints were created between 500 B.C. and 200 B.C. (Benson, 1997).

Wood interlocking joineries hold the members of timber frame tightly in place and transfer building loads to posts then onto the foundation (Lyu et al., 2017). Mortise & tenon joints, according to Peavy and Schmidt (1995), is used to attach two pieces of wood at a right angle (90^o^) using a hardwood peg. Thus, traditional pegged mortise & tenon joints are typically used for post and beam construction of a heavy timber structure. It is therefore not surprising to see countless structures made with mortise & tenon joints erected a few hundreds of years still in use today (Peavy and Schmidt, 1995). It is worth noting that the ancient Chinese people were very good at using wood and created the utilised complex mortise and tenon joints to make wood structures such as beams, struts and roof interlock with perfect fit without using any nails or rivets (Chen et al., 2016). However, this timber-connection method loses popularity in the early 1800s as cheap nails began to replace traditional timber connections (Gold and Rubik, 2009). Then again, this traditional style of connecting is regaining its popularity in housing and heavy timber construction since the 1900s (Bragança et al., 2017). There is a revival of interest in the timber frame construction during the 1970s and since then timber frame has grown to keep up with the increasing demand for the timber houses, barns, and churches (Xin et al., 2017). Mohamed (2014) points out that complicated structural designs limit the utilisation of timber as building materials. The emergence of engineered wood for structural use such as glulam which is a glue-laminated timber has converted timber into versatile building material for wide applications such as large trusses and glulam beams that can span a longer length (Mohamed, 2014). Mortise & tenon joints are the most important parts in the timber frame but also the weakest points (Yeoh et al., 2004). Given the combination of anisotropic behaviour and the nonlinear material properties, the understanding of timber joints increases in complexity (Yeoh et al., 2004). Modern timber frame is energy efficient, comfortable to live or work in, beautiful, and low waste but it remains as a relatively small industry because only very skilled labours are capable to produce mortise & tenon joints making the labour cost much higher than conventional construction (Bergström and Stehn, 2005). The enhancement of automated machines to produce finished timbers is elevating timber productivity and the advancement in technology can definitely make timber frame structures come at more competitive prices (Peavy and Schmidt, 1995). In fact, Yusoff et al. (2008) note that CO2 lasers are often used for the processing (to cut and engrave) of non-metallic materials such as timber simply because these materials have a high absorption of the CO2 laser wavelength. The advantages of compactness, high-reliability, high-efficiency and potentially low-cost of laser materials processing over other mechanical means in producing a successful cut to even thermally sensitive materials such as wood will inextricably shape its future in the field of material processing. Laser cutting has advantages in cutting woods with high accuracy, no tool wear and low noise (Mukherjee et al., 1990). According to Barnekov et al. (1989), the key problem is to understand the complex relationships and interactions between wood and the laser beam. Accordingly, this study seeks to rationalise and demonstrate the efficacy of utilising laser cutting technique in the fabrication of glulam mortise & tenon joints in timber frame.

## 2 A review on mortise & tenon joints and laser cutting

### 2.1 Mortise & tenon joints

Connections entirely made of wood have been used for centuries but their characteristics have never been described mathematically (Brungraber, 1985; Bulleit et al., 1996; Mohamed and Abdullah, 2014). The mortise & tenon method of frame construction consisted of hand-hewn timbers with notched and pegged joints (Soltis et al., 1986). In other words, a tongue on one timber is fitted into a pocket in another, forming a simple, well-fitted and yet strong woodworking joint.

There are many ways to make mortise & tenon joints (McCracken, 1996). Faster ways usually involve the use of power tools such as routers (Lyu et al., 2017). The router bit is guided around a piece of wood to shape the desired tenon (Bulleit et al., 1996; Reynolds et al., 2016; Schmidt et al., 1996). To hand-cut flawless tenons, a tenoning jig can be attached to the table saw (Bragança et al., 2017). Mortises are usually cut in three ways: simply by drilling out the waste and then chopping the mortise square with a chisel, by using a router and a straight bit, or with a hollow chisel mortiser, which is a dedicated mortising machine (Mohamed and Abdullah, 2014; Soltis et al., 1986). Nonetheless, there are pricier jigs and machines in the market that make the process even faster and easier (Xin et al., 2017).

### 2.2 Recent developments in laser cutting

Hilton (1997) observed that gas-aided laser cutting was initially tested in May 1967. These experiments spearhead the exploitation of laser material processing applications. By 1970, laser cutting technology was used in the cutting of titanium rods in aerospace engineering. In recent years, CO2 laser cutters are gaining traction in non-metals such as wood and glass (Shin et al., 2017). Today's laser cutting tools are equipped with high power optics and micropositioning systems that can produce complicated stents as small as 0.002 mm (Madic et al., 2012). Generally, there are three types of cutting that can be performed by the laser cutter, namely: sublimation cutting, fusion cutting, and flame cutting (Yilbas et al., 2017). In laser sublimation cutting, metal evaporating removes the materials. This needs light densities which are obtainable by suitable adjustment of the laser radiation and focusing (Plum et al., 2000). Argon and nitrogen are usually used to prevent the oxidation of the cut edge. Smooth cuts are usually obtained because there is no melting occurs. Laser fusion cutting demands less power because the material is melted and blown out with inert gas jet. In laser flame cutting, pure oxygen is utilized instead of inert gas (Powell, 1993). The material is heated higher than the ignition temperature until a highly exothermal reaction occurs and supplies energy for the cutting process (Liu et al., 2019). Laser flame cutting and laser fusion cutting are predominant in the precision cutting of metals. According to Madic et al. (2012), there are several advantages of laser cutting such as convenient operation, higher accuracy, small heat-affected zone, low waste and low level of noise, flexible operation, ease of automation, high speed, high reliability, narrow widths, easy programmability, cost reduction, minimal material distortion, and versatile nature.

How does laser cutting work on wood? Barnekov et al. (1986) opined that the parameters affecting laser cutting quality for wood can be generally categorised into 3 areas: characteristics of the laser beam, equipment and process variables and properties of the workpiece. Generally, 200 to 800 Watt lasers are used for wood-cutting. However, adjustments are made considering the cutting speed and laser power for maximum efficiency – subject to workpiece thickness, density and the desired Kerf width (amount of material a laser cutter will take away). Types of woods suitable for laser cutting include: alder wood, balsa wood, beechwood, basswood, cedarwood, fine wood, medium-density fiberboard (MDF), natural wood, plywood, solid wood, solid timber (Ng et al., 2000). Wood has been used as a building material for thousands of years and is still one of the most widely used building materials due to its efficiency, durable, and usefulness. Also, wood is highly machinable and can be fabricated into all kinds of shapes and sizes to fit practically any construction need. Being an environmentally sustainable, biodegradable and renewable construction material, harvested wood is a great alternative to non-renewable resources like concrete, steel, and plastics. Notwithstanding the benefits of wood and advances in laser technologies, the use of laser machining of wood is still lacking in the wood industry (Yusoff et al., 2008). Given the current low adoption rate of using the laser cutter to fabricate glulam mortise & tenon joints, this study explored effective of using such technology for rapid manufacturability.

## 3 Experiment design and procedures

**Figure 1:**
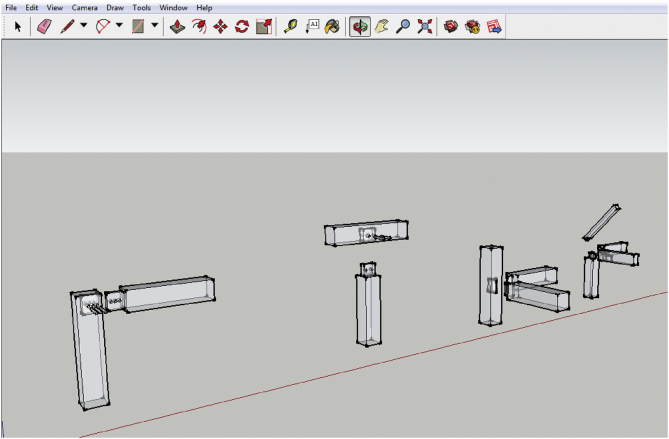
Designs of all 4 models.

**Figure 2:**
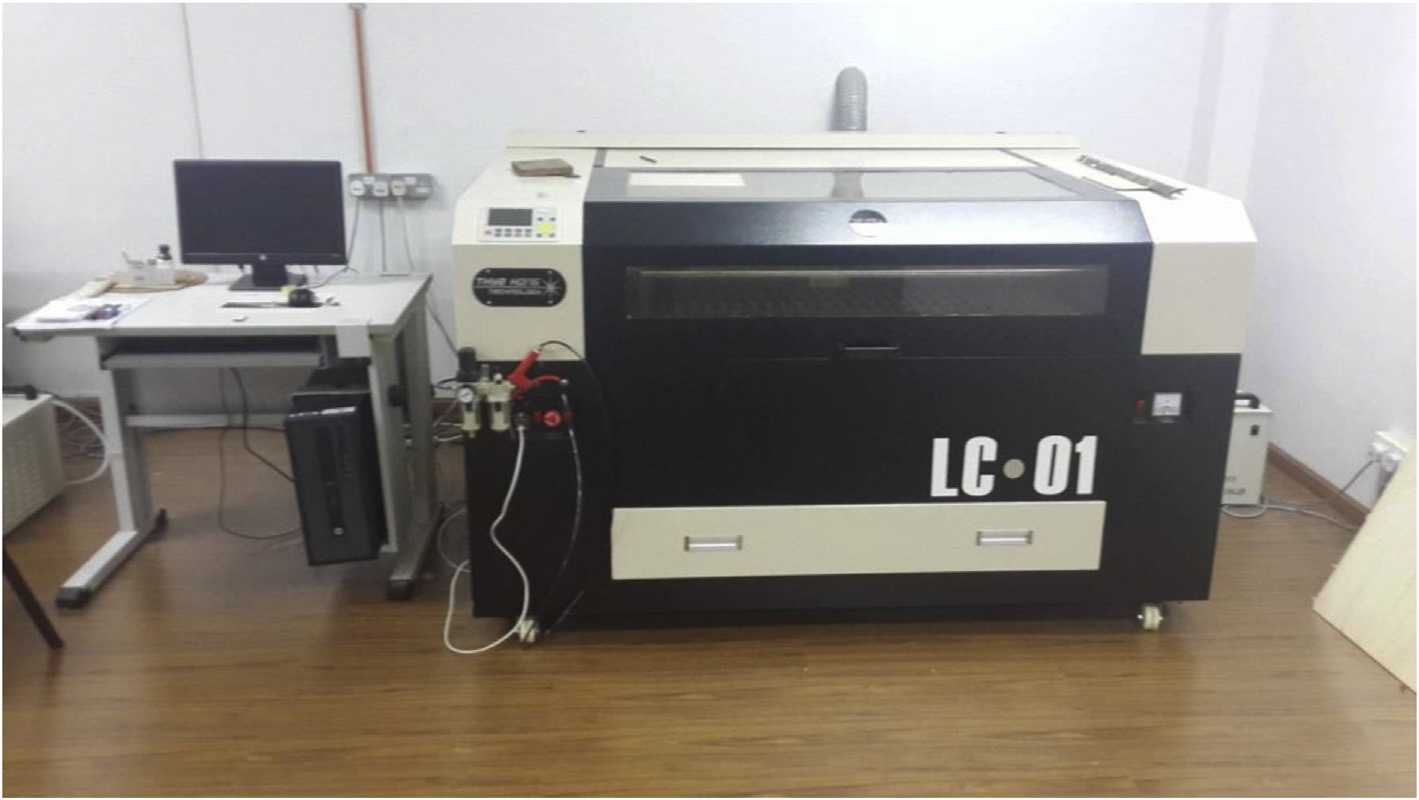
Laser cutter TH 1390/6090.

Trial-and-error experiments aided by laser cutter were conducted to produce 3D timber mortise & tenon joints models. Google SketchUp 2016 is utilised to sketch the 3D mortise and tenon joints. As a result of errors and failures, several files were generated for each model. The sketches were also saved as different files whenever a major amendment was made for better file management. The finalised designs of the models are illustrated in Figure 1. The laser cutter TH 1390/6090 as shown in Figure 2 can cut acrylic up to 25mm thick and plywood up to 12mm with smooth edges and no saw-tooth. It could also fast cut 2mm small circles without deformation. Its specifications are listed in Table 1. Plywood is a type of sheet material manufactured from thin layers of wood veneer glued together with adjacent layers was used in laser cutting and the thickness was 6mm and 9mm. Plywood was chosen because it could produce smooth and accurate cut edges whereby the surface could remain crack-free, and it could increase stability due to its laminated nature. As the laser cutter can only cut plywood up to 3 feet by 4 feet with the maximum thickness up to only 12mm, the dimension of the samples used in the tests is confined to this equipment limitation. Thus, the build size of the 3D model is controlled by the equipment limitation - it shall be few millimetres away from the edges of the plate. In the context of mortise and tenon joints, it must be noted that the tenon's width must not be less than one-third of the thickness of the wood especially if the wood of the same thickness is joined. The shoulders can be of any width. Although the samples were relatively smaller as compared to the actual construction size, the reduce-scale model is apt to demonstrate how the concept of laser cutting can be applied in construction technology and the ways to transform the 3D animated models to the real structure via this modern laser cutting technology.

**Table 1:** Specifications of Laser cutter TH 1390/6090.

Processing area	3 feet × 4 feet
Location precision	±0.05 mm
Repeat precision	0.05 mm
Mat cutting speed	60 m/min
Power laser	60W/80W/100W/150W CO2 glass tube
Blowing system	Lower blowing system (upper blowing system is available)
Optical path	Fly light path
Machine dimension	1820 × 1380 × 1050/1480 × 1120 × 1050

Google SketchUp was employed as the modelling software to design the 3D mortise & tenon joints selected from a pool of applications including: Tinkercad, 3DTin, Autodesk Inventor, Solidworks, Rhino, Creo, AutoCAD, and 3ds Max, due to the reasons that it is free of charge, user-friendly for beginners, and highly compatible with most laser cutters. CAD span Plugin for SketchUp was utilised to aid the production of 3D printable files from SketchUp as well as checking and converting drawing format, which could highlight the problems in a model, easily move all geometry within components and groups to a layer, carry out resurfacing to shrinkwrap a model into a continuous and solid mesh, as well as toggle smoothing on a model's rounded geometry. The CADspan engine produced STL files that describe the exterior of the CAD files ready for 3D printing. MeshLab was also used for final checking of the 3D models and provided an alternative to export the 3D drawings to STL (.stl) format or OBJ, 3DS, PLY, STL, OFF formats, which was used to remove any duplicated faces, automatic filling holes, and smoothing (Wang et al., 2018). Laser CAD v7.52 was used to transfer the 3D models to the laser cutter because it is compatible with AI, BMP, PLT, DXF, DST formats.

## 4 Experiment results and finding

### 4.1 3D modeling on four types of mortise & tenon joints

**Figure 3:**
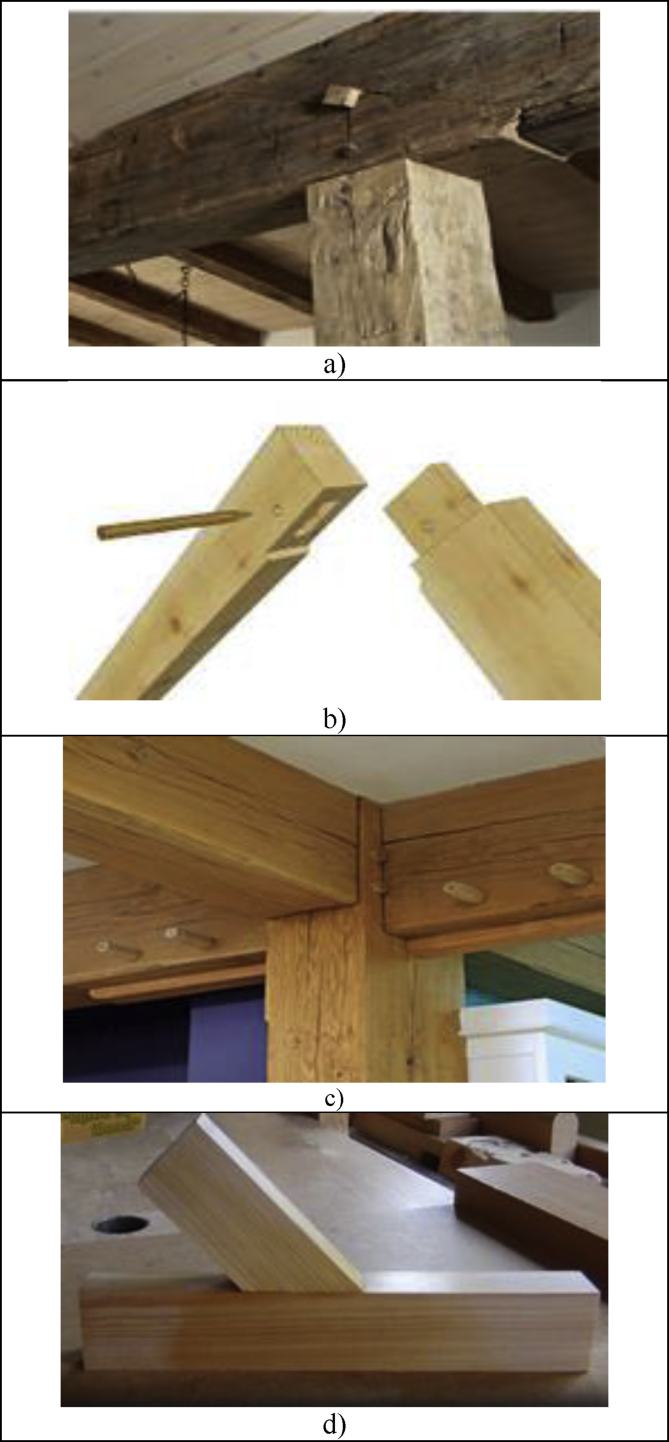
Four types of mortise & Tenon joints used in 3D modelling and laser cutting: a) blind mortise & Tenon joint; b) corner blind mortise & Tenon joint; c) interlocking mortise & Tenon joint; d) hybrid mortise & Tenon joint combining blind, interlocking, and angled.

Four types of mortise & tenon joints including blind mortise & tenon joints, corner blind mortise & tenon joints, interlocking mortise & tenon joints, and hybrid mortise & tenon joint combining blind, interlocking, and angled were used in the 3D modelling as presented in Figure 3. Blind mortise & tenon joint was the joint used to connect blind mortise and stub tenon, and neither was visible at the connected joint. The tenon was fully hidden in the mortise. This type of joint was strengthened with dowels and wedges. Blind mortise & tenon joint is one of the most basic types of mortise & tenon joint which is strong and durable was usually seen in the post and beam construction in the timber frame. Corner blind mortise & tenon joint was pretty similar to the blind one, which was normally applied in the roof to connect two rafters. Interlocking mortise & tenon joint was common in construction to connect beam and column, where there was a concave part at one of the timber beams thus posting a certain level of difficulty to the design and also the fabrication part.

**Figure 4:**
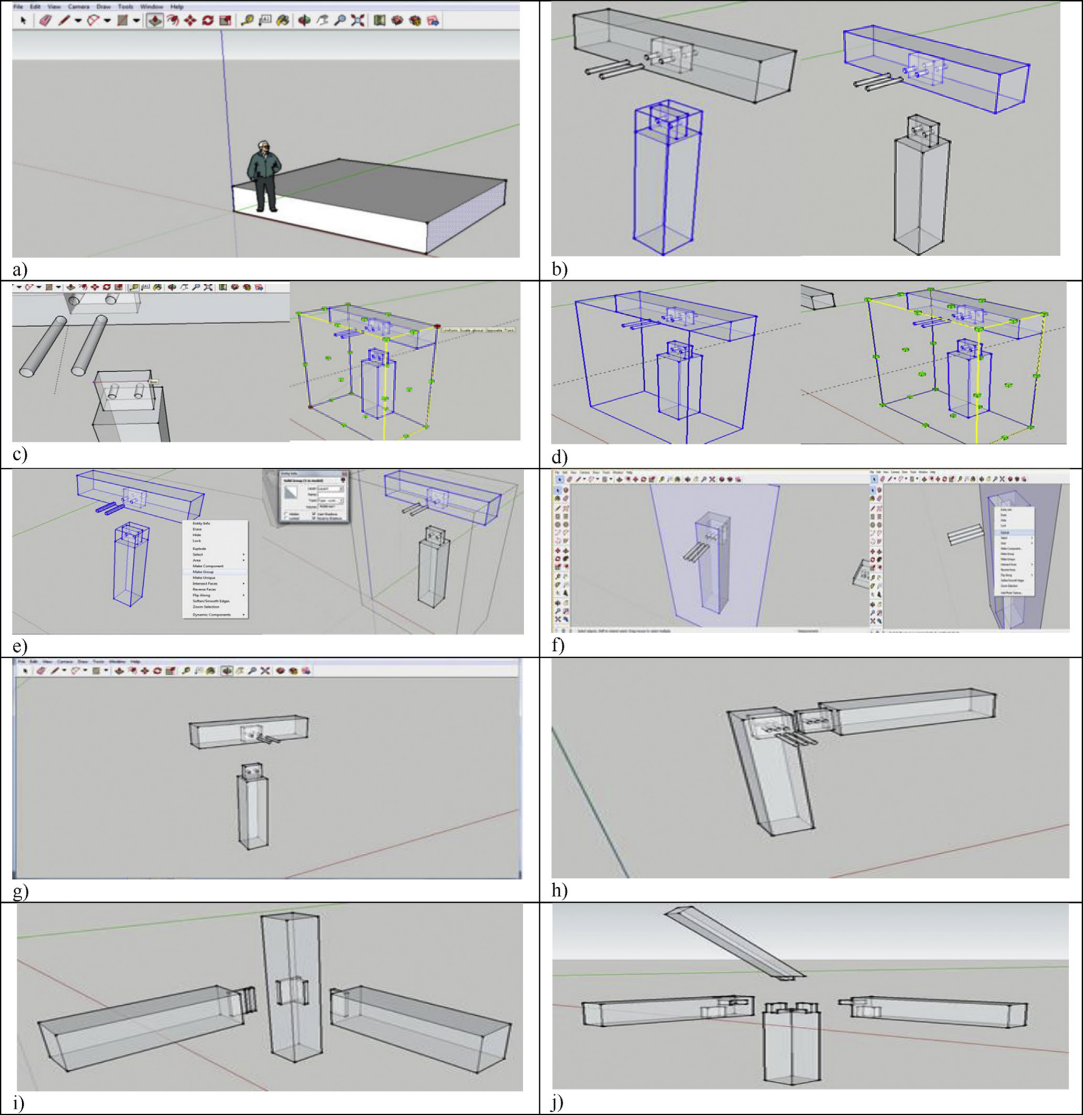
Sketching using Google Sketch Up: a) Draw a plane surface and pull it into 3D; b) Make a particular part into component; c) Checking the dimension of mortise and Tenon; d) Checking the maximum thickness of model; e) Rescaling the dimension of the 3D models; f) Unfolding the structure; g) SketchUp Model 1: Blind mortise & tenon joint; h) SketchUp Model 2: corner blind mortise & tenon joint; i) SketchUp Model 3: Interlocking mortise & tenon joint; and j) SketchUp Model 4: Hybrid mortise & tenon joint.

The hybrid mortise & tenon joint combining blind, interlocking, and angled showcased a higher level of difficulty in designing and fabrication. The tenon's width must not be less than one-third of the thickness of the wood especially if the wood of the same thickness is joined, and the shoulders can be of any width. Figure 4 presents the sketching using Google SketchUp, which includes: a) draw a plane surface and pull it into 3D; b) make a particular part into component; c) checking the dimension of mortise and tenon; d) checking the maximum thickness of model; e) rescaling the dimension of the 3D models; and f) unfolding the structure.

### 4.2 Converting format for laser cutting

**Figure 5:**
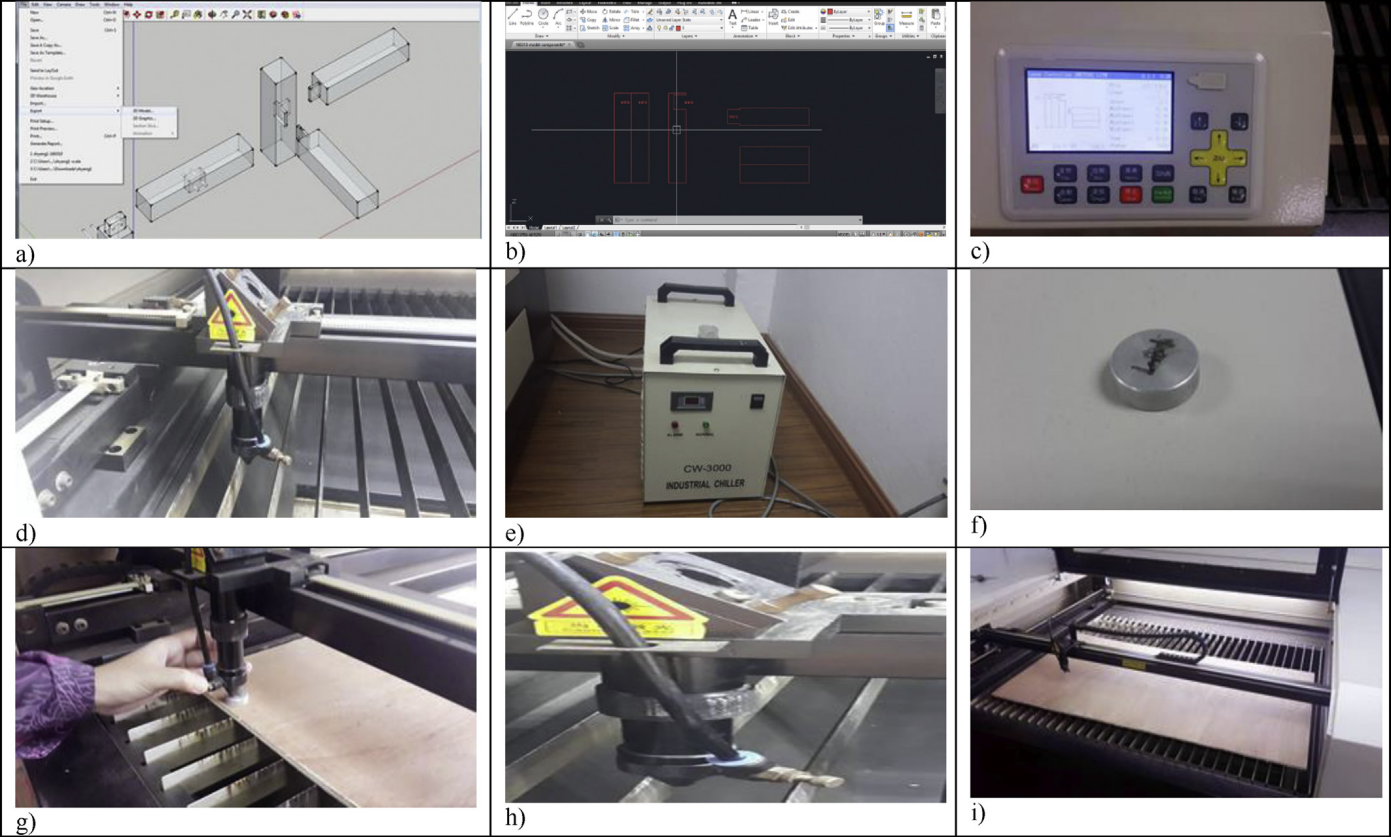
Set up apparatus for Laser Cutting: a) Export as .dxf file; b) Check the file with AutoCAD; c) Send the image to laser cutter; d) Lens cleaning; e) Chiller; f) Round metal for laser head; g) Setting laser head height; h) Blower; and i) Air cut.

Files saved in Google Sketchup was in .skp format ready to be exported to .dxf format through CADspan Plugin so that it could be viewed by the laser cutter software Laser CAD V7. Figure 5 presents the setup apparatus for laser cutting, which includes: a) export as .dxf file; b) check the file with AutoCAD; c) send the image to laser cutter; d) lens cleaning; e) chiller; f) round metal for laser head; g) setting laser head height; h) blower; and i) air cut. The order of cutting was an important parameter because when the outer border of a part was cut, the pieces often “fall out" of the stock material and slightly skew rotationally. Pieces sent to the "back" were cut first and pieces sent to the "front" were cut last. The outside borders of parts were therefore sent to the front. The lens was kept clear and cleans from any dirt because if the laser cutting was done with a dirty lens, particles might ignite and burn to destroy the lens. The chiller was turned on during laser cutting to prevent the laser cutter from over-heating. The chiller was with completed water flow and high-temperature alarm function. The laser head was kept 8.0 mm above the material with the aid of a round steel material as shown in Figure 5 to produce good quality. The blower was turned on to prevent heat and smoke going uncontrolled.

### 4.3 Fabricated glulam mortise & tenon joint

**Figure 6:**
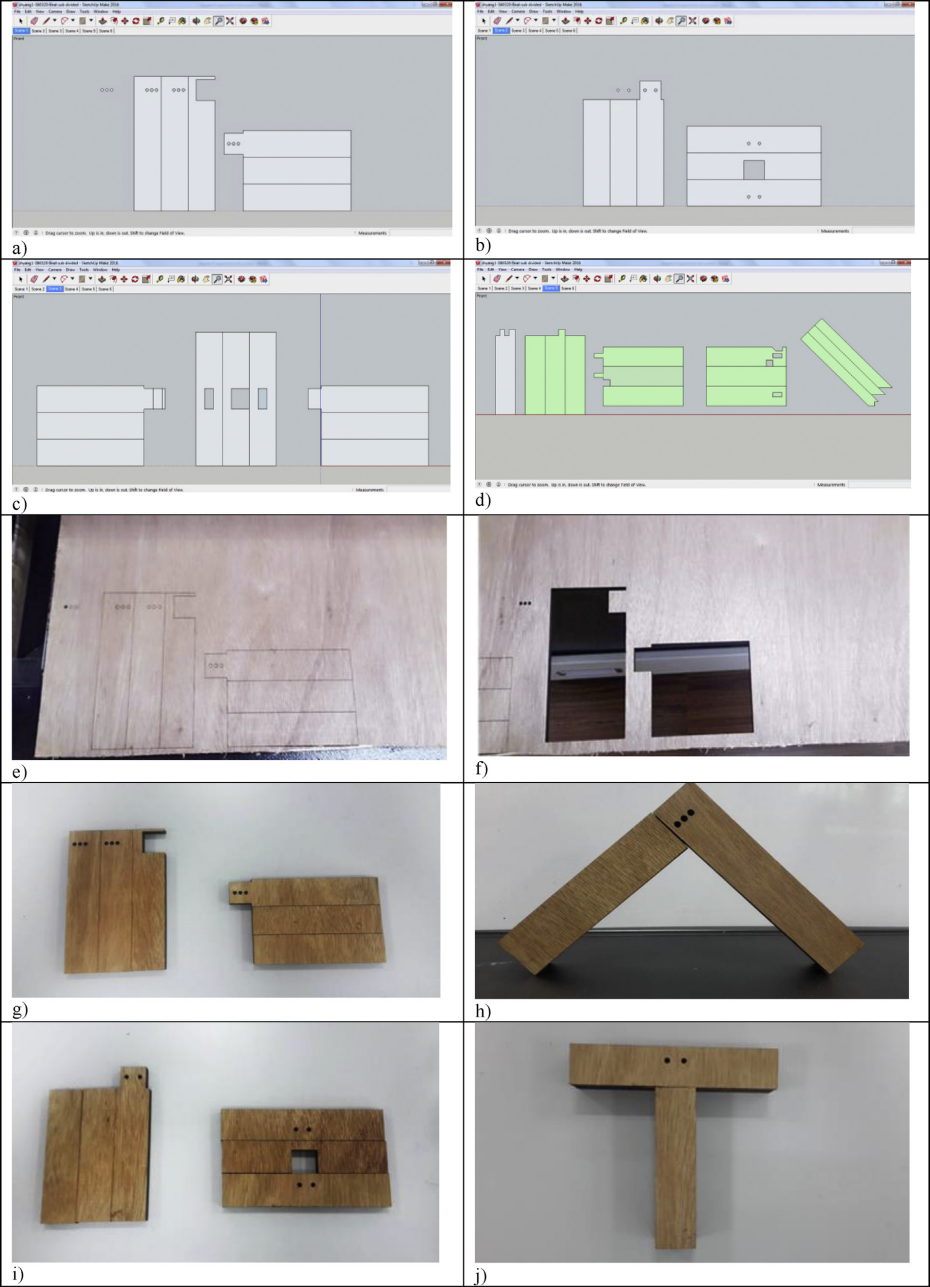
Models in 2D images and Models 1&2 3D-printed: a) 2D images for model 1; b) 2D images for model 2; c) 2D images for model 3; d) 2D images for model 4; e) drawing for Model 1; f) Model 1 hollow out; g) Model 1 before assembling; h) Model 1 assembled; i) Model 2 before assembling; and j) Model 2 assembled.

**Figure 7:**
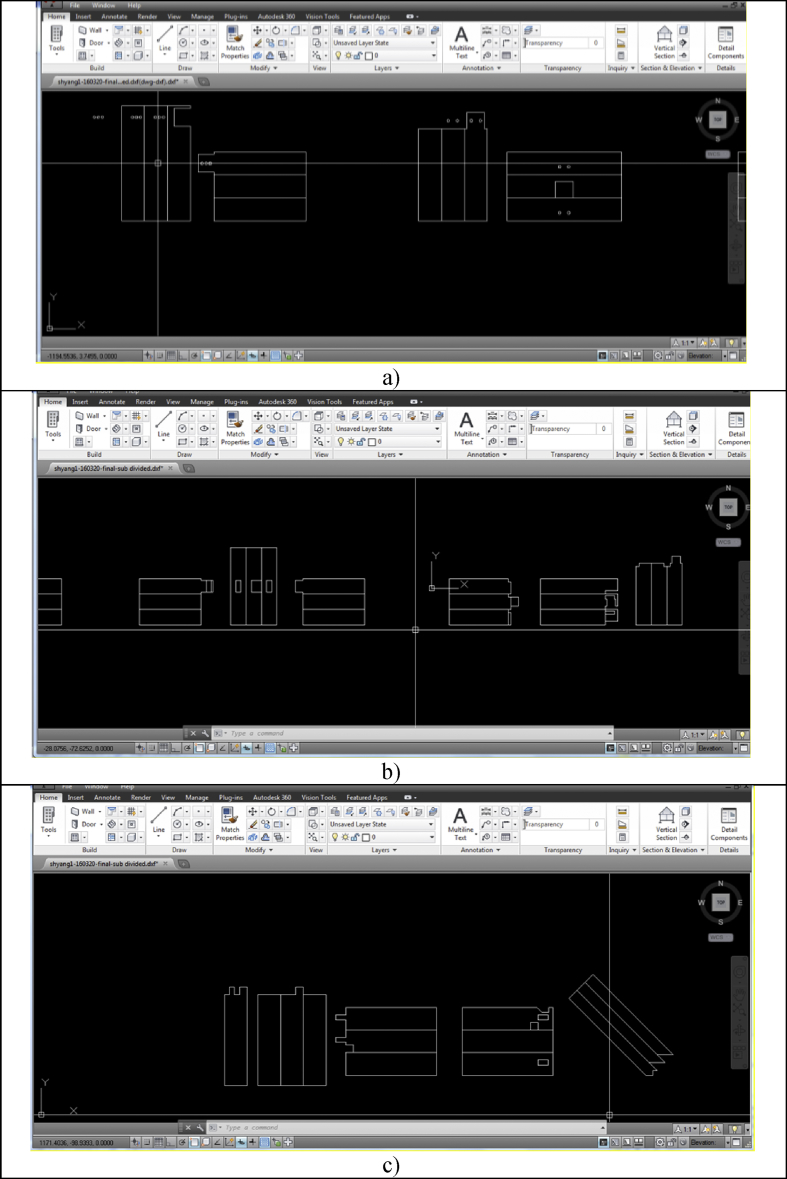
AutoCAD files. a) 2D image for Models 1 and 2; b) 2D image for Model 3; and c) 2D image for Model 4.

**Figure 8:**
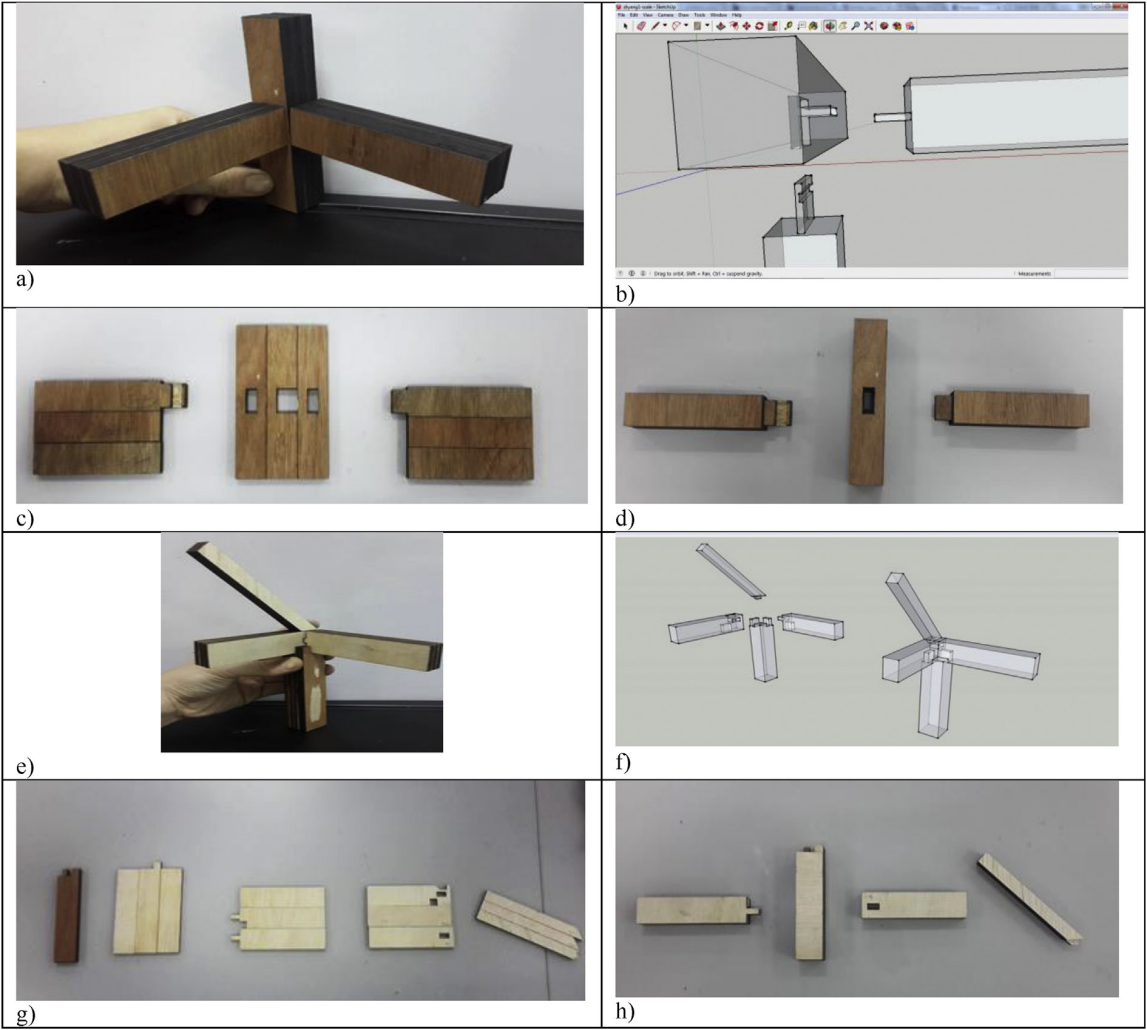
Model 3&4 laser cut and assembled: a) Model 3 assembled; b) Concave part of Model 3; c) Model 3 laser cutting products; d) Model 3 products after glue-ing; e) Model 4 assembled; f) Design of Model 4; g) Model 4 laser cutting products; and h) Model 4 products after glue-ing.

After creating all structures in the 2D forms using Google SketchUp, files were exported in .dxf to layer the images with Auto CAD. The laser cutter could not cut 3D images directly but two-dimensional images instead. Each layer was set to different cutting configurations. The power and speed were set into 100 and 10 respectively. Figure 6 presents models in 2D images and models 1&2 produced whereas Figure 7 exhibits the 2D images in AutoCAD format. Figure 8 presents models 3 & 4 laser cut and assembled. Models 1, 2 & 3 were comparatively simpler to produce than the Model 4 that took several times of trials to succeed.

In Model 4, the structure had one part with two lines to be cut to the depth of 4.5mm instead of 9.0mm. We layered the structure by colours to denote ‘cut-in’ in green and ‘cut-out’ lines in black, whereas the engrave part in blue. The laser cutter could not cut the concave and convex parts directly but to unfold the structures and a chisel was needed. When interacting with plywood, the CO2 laser had a better energy density in comparison to YAG laser.

**Figure 9:**
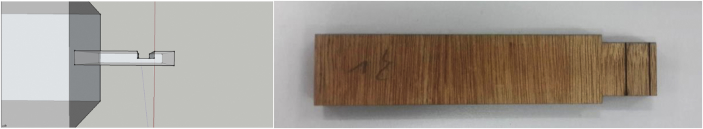
Concave part of hybrid mortise & tenon joint combining blind, interlocking, and angled in Model 4.

The laser-cut components resembled the final products named glulam or glued laminated timber were the modern way of manufacturing timber structures when natural solid-sawn timber could not meet the large size and unusual shape. Before glueing, the laminates were dressed to an exact and uniform thickness and clamped together under constant pressure until the glue cured. Because the glulam was made up of laminates, strength-reducing characteristics were absent or just confined to one laminate and as a result, the product was stronger and more reliable than solid timber. Layering did not affect the strength of the timber structure. In recent years, glulam being a structural engineered wood product has emerged as a versatile, durable and flexible building material fit for a wide variety of applications ranging from large flat roof systems to complex arches meeting demanding environments of bridges, utility poles, cross arms, and marinas. In this regard, gluman systems are frequently specified for its strength, beauty and reliability; providing a viable alternative to structural concrete and steel as glulam can produce a lighter weight structure with a lesser carbon footprint that is much stronger, pound-for-pound. Building advancements using this material have drastically changed the scale of what is architecturally possible using timber in the built environment. The glulam structures have the potential to span a longer span with no intermediate posts or columns. There was no need for false ceilings to cover the structural framework using glulam. Moreover, with the use of laser cutting, the production could be accurate and fast thus reducing the need for on-site fabrication. Glulam is a sustainable building component with low carbon footprint because it does not need to be mined or subjected to high energy demand manufacturing processes compared to steel and cement. Termite resistant materials such as naturally resistant timber or treated timber could be used as roof trusses, lintels, top plates and bottom plates to minimise the termite damage, and the treatment could be done using chemicals such as boron or arsenic. The laser cutter could not cut the 3D parts such as concave or convex parts directly and some layering and resembling work was needed as shown in Figure 9.

The power represents how strong the laser fires on the surface of the materials. Higher the power, stronger the laser cut through the materials. The speed represented how fast the laser cutter moved along its pate. Higher the speed, the faster laser cutter glided along its path, but it might not be able to cut all the way through when the materials were over thick. Lower speed might cut through, but it might burn the edges of the materials. Depending on the workpiece thickness, density and the desired kerf width, the laser cutting parameters (cutting speed and laser power) can then be tuned. Kerf refers to the width of material that is removed by a cutting process. This end, laser cutting kerf is the amount of material the laser burns away. Advantages of laser technology are high processing speed, small kerf, very small heat-affected zone and minimum shrinkage of the cut material as compared to other thermal cutting processes. Laser cutting produces a tapered Kerf profile. The channeling effect of a focused beam penetrates the material better to produce wider kerf with less taper. Kerf width variation during cutting depends on many factors, primarily on laser power, focus settings and cutting speed. Laser cutting parameters are adjusted according to workpiece thickness, density and the desired Kerf profile - increasing laser power intensity and thicker sheet result in wider Kerf size. Cutting using laser power of 800 W: Kerf width is 0.2 mm for sheet thickness of 1 mm, 0.25 mm for 3 mm, and 0.3 for 6 mm. In addition, the cut quality is also influenced by the perpendicularity and slant tolerance. At a constant cutting speed, wet wood required more cutting power as compared with dry wood. To ensure successful cutting with the laser cutter, no double lines were allowed in the drawing. Laser cutter produced clean and precise timber edges and there was no need to use sandpapers. The laser cutting accuracy was extremely superior according to the design, and it offered uniformity from component to component. The laser cutter performed the works that were normally performed by a group of conventional tools such as cutting, drilling, drilling at an angle, marking, and engraving; delivering both labour- and time-efficient procedure. Oxidation led to the darkening of the products’ edges because of the exothermic reaction and the cutting with the compressed air showed serious burn and charring such as carbon residue. The edge darkening depended on the content of resin and water in the materials and the basic rule is that the dryer and free of resin the materials are, the cutting edges are brighter. Unlike the traditional cutting methods where woodworkers need to hold the wood piece in place while cutting, there is no need to do so with laser cutting.

## 5 Conclusions

The usage of timber in structural members attracts much less confidence than non-structural members in modern construction due to the misconception that timber is not as strong as steel, masonry and concrete, and it requires connections made of other materials, yet glulam mortise & tenon joints greatly enhance the usage of timber work. Precision laser cutting supports easy automation, produces small heat-affected zone, minimises deformity, relatively quiet and produces a low amount of waste. Besides, laser cutting is cost-effective and low risk of material contamination. Then again, conventional mechanical cutting involves contamination and deterioration of blades whereas the laser is free of wearing out. LaserCAD could not process 3D images directly but 2D images, so layering and unfolding works were needed before sending to the laser cutter. From the experiments conducted, the products were found to resemble glued laminated timber (glulam) and believed to have the potential to be used as the structural member in construction. Glulam offers large section sizes, long spans, and a high degree of dimensional stability. Notably, glulam mortise & tenon joints are resistant to general corrosion attack. Accordingly, this study demonstrated a significant potential of rapid manufacturability of mortise & tenon joints with high-quality and high-uniformity through computer-aided laser cutting technique for wide applications in the built environment.

This study is not without its limitations. The development of 3D timber frame members was established solely using Google Sketchup by choice without deliberating alternative CAD platforms. It should be cautioned that only one type of material was used in the experiment, which was plywood. As such, the laser cutting on the other types of wood is recommended for future study. Given that each type of material will require different speed and power to attain the desired cut depth, some indexes may possibly be developed to guide laser cutting on different materials and with different laser cutting instruments.

## Declarations

### Author contribution statement

Zhaoxi Zhan: Conceived and designed the experiments.

Chen Wang: Performed the experiments; Wrote the paper.

Jeffrey Boon Hui Yap: Analyzed and interpreted the data.

Ming Shyang Loi: Contributed reagents, materials, analysis tools or data.

### Funding statement

This work was supported by the Quanzhou Science and Technology Bureau under Grant [number 2018Z010]; and the work was also supported by the Fujian science and technology department under Grant [number 2019R0057]; and the work was also supported by the 10.13039/501100003815Huaqiao University under Grant [number 17BS201].

### Competing interest statement

The authors declare no conflict of interest.

### Additional information

No additional information is available for this paper.
